# Improving health evaluations to capture wider value of therapeutics and incentivise innovation

**DOI:** 10.3389/fpubh.2023.1119652

**Published:** 2023-03-31

**Authors:** Mei Sum Chan, Jack C. Kowalik, Tom Ashfield, Jonathan Pearson-Stuttard

**Affiliations:** ^1^Health Analytics, Lane Clark & Peacock LLP, London, United Kingdom; ^2^UK Health & Value, Pfizer Ltd., Tadworth, United Kingdom; ^3^UK Medical Affairs - Anti-infectives, Pfizer Ltd., Tadworth, United Kingdom; ^4^Department of Epidemiology and Biostatistics, Imperial College London, London, United Kingdom

**Keywords:** elements of value, insurance value, antimicrobial resistance, pandemics, value assessment

## Prevention is better than cure—but how to value prevention and incentivise investment?

The health of the population is inextricably linked to wider economic prosperity, and COVID-19 has brought this into sharp relief (1). With deaths due to COVID-19 reaching 7 million worldwide (2), there has never been a more pivotal time to call for greater protection against future health threats. However, one of the greatest challenges is recognizing and quantifying the full value of prevention and preparedness to patients, health systems and society. Any such valuation must be comprehensively estimated to include not only the adverse consequences avoided, but also the wider benefits of effective interventions. This will align incentives to invest in patient and population health.

The COVID-19 pandemic has starkly revealed how interventions that prevent illness and maintain good health, such as vaccines, antimicrobials and antivirals, provide value beyond the healthcare system alone. Not only do they alleviate illness, they also mitigate disease transmission, protecting the wider population and enabling education, work, caring and social interactions to continue. Prevention of non-communicable diseases such as heart disease and diabetes also confer similar types of value. However, traditional approaches to assessing the effectiveness of such interventions rarely capture value added beyond the healthcare setting. Something needs to change.

## Realigning incentives to promote investment in population health priorities

By viewing through a lens of health being an asset rather than illness being a cost, the healthcare system could promote health in communities, rather than paying for treatment of ill health. Unfortunately, neither good health nor the resilience across health systems that this would support are commonly valued or incentivised (3).

There are signs of progress. NICE is exploring wider definitions of value (4, 5), and its latest guidance on antimicrobial resistance (AMR) is designed to reward innovation and de-link payment from quantity sold (5, 6), to support appropriate use of the antimicrobials. This echoes recent research that found therapeutic benefit was most commonly regarded as a measure of innovation (7). Secondly, the societal impacts of vaccines (8) and antivirals (9) were an important consideration during the pandemic. Thirdly, there is increasing appetite for Outcomes Based Agreements to better align commercial arrangements around patient value. Finally, broader frameworks for valuing novel antimicrobials and vaccines (3, 5, 6, 10–12) have also been recommended internationally.

## Gaps in approaches for assessing value

Health Technology Assessments (HTAs) use a standardized approach to assess population-level benefits and the comparative value of interventions such as therapeutics and screening programmes. However, these assessments focus on single diseases, despite multimorbidity now being the norm for many patients. Traditional health economic perspectives also exclude cost savings to wider health and community services (e.g., releasing capacity in other facilities), and indirect economic and societal benefits (e.g., improving patients' ability to work) (13). These may substantially outstrip the benefits to health services (13). This situation often leads to health interventions being systematically undervalued (3).

There are no established methods for assessing broader value, despite numerous recommendations to consider them for novel antimicrobials and vaccines (3, 10–12). Key elements of this broader value include insurance value and enablement value (3, 6, 10, 14).

Insurance value is the value of having a treatment available in case of future major or rapidly escalating health problems, while enablement value is the value of enabling other treatments or procedures to go ahead (14). Insurance value, for instance, is commonly assessed in the insurance sector (15) to quantify the prevention or mitigation of financial risks, terrorism, cyber incidents and natural disasters. It is also assessed to determine the resources to hold in reserve, from either a risk-neutral or risk-averse perspective. Sophisticated risk prediction models assess frequency and severity patterns of potential adverse impacts and aggregate them across all relevant risk events, not just the most catastrophic events. These can be adapted to the health context.

Adverse health events such as COVID-19 and a catastrophic increase in AMR could lead to disruptions similar to a natural disaster, with dramatically higher mortality and economic downturns (16). Deaths associated with bacterial AMR have surpassed 4 million in 2019 alone (17). A first step has been taken with the guidance on evaluating broader value of antimicrobials (5). However, methodological approaches for valuing the mitigation of multiple risk events, assessing value beyond recouping development costs and evaluating multimorbid patient pathways, are needed.

To bridge this gap, we need focused and coordinated action across two areas. First, research must quantify insurance value and broader value elements specific to each therapeutic area, and characterize how this value changes over time and across population groups. Second, HTAs of therapeutics must advance beyond current approaches toward holistic evaluations representative of both patient need and population health value, acknowledging that different models may be required for different therapeutic areas and health systems.

## Conclusion

Valuing health holistically and investing in innovation and health system resilience could help tackle the biggest health challenges of the 21st century. The COVID-19 pandemic has increased awareness of the wider impacts of health and illness on society, while aging and multimorbid populations will put greater burdens on healthcare systems in future years. Research and policies from regulators, academia and industry must enable health systems to incorporate holistic value assessments used by other sectors into HTAs, and re-invigorate investment in healthcare innovation (6, 7). This will realign incentives and recognize the complexity in maintaining health beyond treating specific illnesses.

## Author contributions

All authors contributed to the drafting and review of the manuscript, and read and approved the final manuscript.
